# Distinct variations of antibody secreting cells and memory B cells during the course of Kawasaki disease

**DOI:** 10.1186/s12865-019-0299-7

**Published:** 2019-06-03

**Authors:** Meng Xu, Yanfang Jiang, Jinghua Wang, Jinxiang Liu, Congcong Liu, Deying Liu, Sirui Yang

**Affiliations:** 1 Department of Pediatric Rheumatology, Immunology, and Allergy, The First Hospital of Jilin University, Changchun, 130021 China; 2 Genetic Diagnosis Center, The First Hospital of Jilin University, Changchun, 130021 China; 3 Jiangsu Co-innovation Center for Prevention and Control of Important Animal Infectious Diseases and Zoonoses, Yangzhou, 225009 China; 4 Wuhan Children’s Hospital, Tongji Medical College, Huazhong University of Science & Technology, Wuhan, China

**Keywords:** Kawasaki disease, Antibody secreting cells, Cytoplasmic IgG, Memory B cells

## Abstract

**Background:**

Both antibody secreting cells (ASCs) and memory B cells are essential for the maintenance of humoral immunity. To date, limit studies have focused on the two populations in Kawasaki disease (KD). To address the status of humoral immunity during KD, our current concentration is on the variations of ASCs and memory B cells, as well as their subsets in both acute and remission stages of KD.

**Methods:**

ASCs were defined as the population with high expressions of CD27 and CD38 among CD3-CD20- lymphocytes. Based on the expression of surface marker CD138 and intracellular marker IgG, ASCs were further divided into two subsets. Memory B cells were characterized by the expressions of IgD, CD27 and IgM, upon which memory B cells were further categorized into CD27 + IgD- (switched memory, Sm), CD27-IgD- (Double negative, DN) and CD27 + IgD + IgM+ (marginal zone, MZ) B cells. Collectively, six populations were analyzed using flow cytometry. The blood samples were collected from KD patients in different stages and healthy controls.

**Results:**

In the acute stage, the percentages of ASCs, CD138+ ASCs, and IgG+ ASCs were significantly increased. In contrast, the percentages of memory B cells including Sm and MZ B cells were significantly decreased. Correlation analysis found ASCs positively correlated with the level of serum IgM, whereas MZ B cells not only positively correlated with the level of serum IgG, IgA, and IgM, but also positively correlated with the level of serum complement C3 and C4 and negatively correlated with the value of C-reactive protein (CRP). In the remission stage, the percentages of IgG+ ASCs and MZ B cells were significantly reduced, whereas other subsets presented heterogeneous variations.

**Conclusions:**

Our study provided direct evidence that ASCs contributed to the pathogenesis of KD, and it was the first time to describe the variation of memory B cells in this disease. Among the subsets, only IgG+ ASCs presented a significant increase in the acute stage and decreased after IVIG administration, indicating the involvement of IgG+ ASCs in the inflammation of KD and also suggesting that IVIG played an inhibitory role in the expression of cytoplasmic IgG.

**Electronic supplementary material:**

The online version of this article (10.1186/s12865-019-0299-7) contains supplementary material, which is available to authorized users.

## Background

Kawasaki disease (KD) is an acute, self-limited, febrile vasculitis that predominantly affects children under 5 years of age. KD remains the leading cause of acquired heart disease during childhood. Classically, it can be characterized by high spiking fever persisting for more than 5 days, erythematous rash, bilateral conjunctivitis, congestive oral mucosa, swelling lymph node, and edematous extremity [1]. Precisely due to these highly identifiable clinical manifestations, KD is also known as mucocutaneous lymph node syndrome. Intravenous immunoglobulin (IVIG) is the most effective therapy for the improvement of symptoms and the prevention of coronary abnormalities [2]. During the past four decades, investigations on the pathogenesis of KD have never been ceased. Nowadays, aberrant immune responses triggered by invading pathogens on the genetically susceptible individual is thought to be the key point in the occurrence and development of KD [3, 4].

The most visualized immunological abnormality in KD is the activation of innate immunity represented as the elevations of neutrophils, C-reactive protein (CRP), erythrocyte sedimentation rate (ESR), and cytokines such as interleukin (IL)-6, tumor necrosis factor alpha [5]. However, innate and adaptive immunity are interconnected. Innate cells can drive the initiation of adaptive immunity [6]. Indeed, the humoral immunity, which is an indispensable part of adaptive immunity, is demonstrated to participant in the pathogenesis of KD by accumulative evidence. Early studies have shown elevated levels of serum immunoglobulins and activated polyclonal B cells [7, 8]. Increased immunoglobulin complex in circulation has also been reported [9]. Besides, decreased circulating IgA+ B cells and plasma cells were detected possibly due to the infiltration of IgA+ cells into vascular tissues, including the proximal cardiac tract, pancreas, kidney and coronary artery [10, 11]. Recently, researchers found the increased percentage as well as absolute number of CD19+ cells in the peripheral blood of patients with KD [12]. In addition to these direct evidence, our latest study presented an increased level of one activated subset of T follicular helper cells and serum IL-21 [13, 14], which are vital to B-cell proliferation and differentiation [15], strongly suggesting the involvement of humoral immunity in KD. Moreover, with the development of genomics, susceptible genes associated with B cells have also been identified [16]. Therefore, it is convinced that humoral immunity plays a crucial role in KD.

B cells are one of the most important molecules of humoral immunity due to their irreplaceable ability in antigen presentation, cytokines secretion and antibodies production. B cells are comprised of heterogeneous subpopulations with distinct phenotypes. Among these subsets, antibody secreting cells (ASCs) and memory B cells are the key feature and contributors to maintain the humoral immunity. ASCs can be distinguished using flow cytometry based on the bright expressions of CD27 and CD38 in bone marrow, as well as in circulation [17]. ASCs are well-differentiated and effector cells that are responsible for the abundant secretion of antibodies that can neutralize foreign antigens [18]. Despite such obvious advantages of ASCs in human health, excessive or persistent ASCs would lead to pathogenic conditions [19]. In KD, a case report has described a patient with excessive plasmablasts [20]. Another study has reported that the median percentage of plasmablasts in KD patients were 2.51% of circulating B cells, indirectly indicating that the plasmablasts would be increased in KD because the normal level of plasmablasts should be less than 1% of circulating B cells according to the results from the literature [21]. To some extent, the two studies substantiated the involvement of ASCs in KD. However, the exact difference between KD patients and healthy individuals, and their potential roles during KD remain to be determined.

Memory B cell is another crucial component of humoral immunity. Human memory B cells in circulation can be characterized by the expression of their surface markers such as CD27, IgD and IgM among the mature B cells [22, 23]. These markers divide memory B cells into distinct subgroups. Remarkably, although the most of memory B cells are thought to be derived from the germinal centers (GCs) of the secondary lymphoid organs when the encountering with pathogens for the first time [24], the origin of CD27 + IgD + IgM+ B cells remains controversial as a result of their diversified roles in both innate and adaptive immunity. Unlike ASCs, memory B cells are absent of the ability in secreting antibodies, while if the host is re-stimulated, will memory B cells undergo the differential process into ASCs and provide faster humoral response than naïve B cells do, demonstrating that they are of essence for the secondary immune response [25, 26]. To our knowledge, investigation on memory B cells in KD remains to be completed. Nonetheless, the self-limiting nature and low recurrence rate strongly imply the connection between memory cells and KD. Therefore, memory B cells were also taken into this study. Indeed, we found some significant variations in both ASCs and memory B cells. We hope our study would be beneficial for the further understanding of humoral immunity in KD.

## Methods

### Patients

We enrolled 18 KD patients hospitalized in the Department of Pediatric Rheumatology and Allergy, The First Hospital of Jilin University, China, from January to December 2018. After a detailed physical examination and necessary laboratory tests, the diagnosis was established meeting with the criterion that the 2017 American Heart Association (AHA) clinical guidelines [1]. All patients underwent the same treatment options, which were the administration of IVIG at a dose of 2 g/kg for 1 day and oral aspirin at a dose of 30-50 mg/kg per day from the establishment of diagnosis to defervescence. After IVIG administration, all patients were in remission, at which stage the patients had been afebrile for at least 48 h. Eighteen blood samples from patients in the acute stage were collected, whereas only nine of the patients agree with blood collection in the remission stage. Another fifteen sex- and age-matched healthy children who came for healthy examination were chosen. These children had not suffered from any diseases at least in the previous month. Neither patients nor healthy controls had been diagnosed with any autoimmune diseases. Patients with incomplete or refractory KD were not brought into this study. The documented clinical parameters from both patients and healthy controls included: white blood cell counts, neutrophil counts, lymphocyte counts, serum C-reactive protein CRP, ESR, serum immunoglobulins (IgG, IgA, IgM) and complement C3 and C4. The Ethics Committee of The First Hospital of Jilin University authorized the experimental protocol following the guidelines of the Declaration of Helsinki. Written informed consent was obtained from the parents of all individuals.

### Flow cytometric analysis

In order to the successful acquirement of ASCs, four-milliliter fresh blood samples were collected from both healthy controls (HCs) and KD patients in acute and remission stages. Peripheral blood mononuclear cells (PBMCs) at 4 × 10^6^/ml were isolated from each individual by density-gradient centrifugation using Ficoll-Paque Plus (Amer- sham Biosciences, Little Chalfont, UK) at 800×g for 30 min at 25°C. PBMCs were stained with antibodies (Becton Dickinson, San Jose, CA, USA) including surface CD3 (BV510), CD19 (APC-H7), CD20 (BV421), CD27 (PE-Cy7), CD38 (APC), CD138 (PE), IgD (PE-CF594), and IgM (BV515) at room temperature for 30 min. Next, the cells were fixed, permeabilized, and intracellularly stained with IgG (Becton Dickinson, San Jose, CA, USA). Finally, PBMCs were analyzed by multicolor flow cytometry (FACSAria II; BD Biosciences, Franklin Lakes, NJ, USA), and the results were analyzed using FlowJo v10.0.7 software (Tree Star, Ashland, OR, USA).

### Statistical analysis

The data were represented as the median and range and performed with SPSS version 22.0 software. Kruskal-Wallis test was applied to assess the difference among groups. The correlation analysis was evaluated using Spearman’s rank correlation test. The difference between the acute and remission stage was analyzed by the Wilcoxon matched pairs test. *P* < 0.05 was considered to be statistically significant.

## Results

### Patients characteristics

Eighteen KD patients and fifteen healthy children were involved in the present study. Nine samples of KD patients in the remission stage were collected. Their demographic and clinical parameters are shown in Table 1. The differences in age and sex between the KD patients and the HCs were not significant. The patients in the acute stage shown a significantly higher number of white blood cells and neutrophils than those in HCs and the patients in the remission stage, however, their lymphocyte counts did not change obviously. The levels of CRP and ESR were significantly elevated in the acute stage of KD. After treatment, CRP decreased rapidly to an almost normal level, whereas the level of ESR remained higher. The median level of serum immunoglobulins and complement proteins was maintained within the normal range in the acute stage of KD.

**Table 1:** The demographic and clinical characteristics of the study participants

Parameters	Kawasaki disease		Controls (*n* = 15)
	Acute stage (*n* = 18)	Remission stage (*n* = 9)	
Age, year	3.1 (0.9–4.8)	2.7 (1.1–4.8)	3.0 (0.8–5.1)
Sex, Female/Male	8/10	3/6	7/8
WBC, 10^9^/L	15.6 (6.99–33.2) ^†,‡^	7.25 (5.71–10.82)	7.69 (5.1–9.83)
Neutrophils, 10^9^/L	10.35 (1.2–30.8) ^†,‡^	3.18 (1.96–5.5)	2.82 (2.51–5.02)
Lymphocytes, 10^9^/L	3.36 (1.3–8.9)	2.97 (1.87–3.91)	4.06 (1.03–5.3)
CRP, mg/L	49 (12.71–186)^†,‡^	11.5 (3.28–17.1) ^†^	1.7 (0.5–3.8)
ESR, mm/h	75 (12–110) ^†^	79 (15–99) ^†^	6 (2–19)
IgG, g/L	6.485 (1.83–16.3)	ND	5.93 (2.12–10.72)
IgA, g/L	0.83 (0.11–1.29)	ND	0.92 (0.5–2.4)
IgM, g/L	1.045 (0.18–1.77)	ND	1.115 (0.2–1.63)
C3, g/L	1.26 (0.72–2.07)	1.1 (0.63–1.98)	1.32 (0.63–1.8)
C4, g/L	0.275 (0.16–0.51)	0.26 (0.16–0.5)	0.235 (0.18–0.49)

Data are represented as median (range) or the number of cases

*WBC* white blood cell counts, *CRP* C-reactive protein, *ESR* erythrocyte sedimentation rate, *Ig* immunoglobulin, *C3* complement component 3, *C4* complement component 4, *ND* not determined

^†^*P* < 0.05 vs. the controls. ^‡^*P <* 0.05 vs. the remission stage

### The levels of ASCs in different stages of KD

**Fig. 1:**
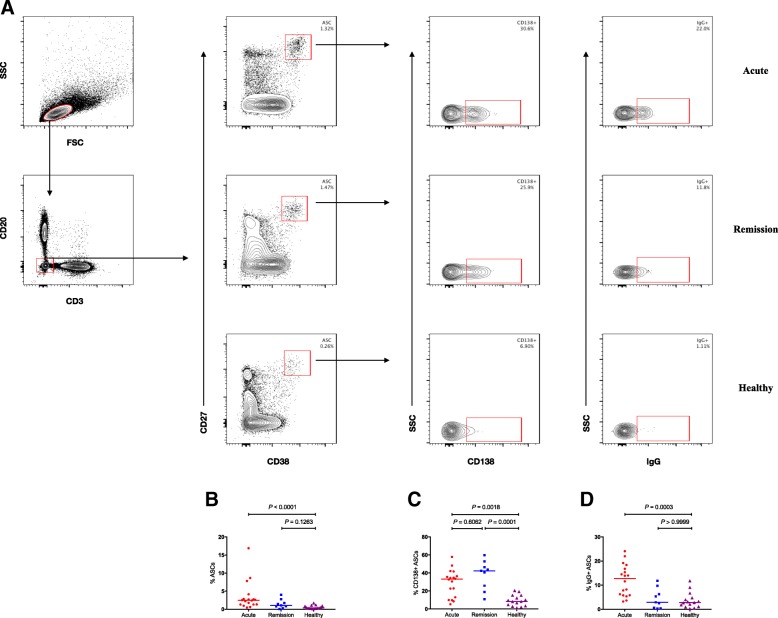
Flow cytometry analysis of the percentage of antibody secreting cells in KD patients. **a** Flow cytometry analysis. **b–d** Quantitative analysis. The data are represented as dot plots or are expressed as the mean percentage of antibody secreting cells of individuals. The horizontal lines stand for the median values. ASCs, antibody secreting cells

To investigate the status of humoral immunity, we examined the overall levels of ASCs and their subsets in both acute and remission stages. PBMCs from all participants were immune-stained with CD3, CD20, CD27, CD38, CD138, and intracellular IgG, and subsequently analyzed by flow cytometry [17]. ASCs can be distinguished by high expression of CD27 and CD38 in the CD3-CD20- lymphocyte. CD138+ ASCs and cytoplasmic IgG+ ASCs were further identified on ASCs gate. The gating strategy of ASCs and their subsets was shown in Fig. 1a. The results shown that the percentages of ASCs, CD138+ ASCs, and IgG+ ASCs were all increased in acute stage of KD (*P* < 0.0001, Fig. 1b; *P* = 0.0018, Fig. 1c; *P* = 0.0003, Fig. 1d; respectively). Therefore, our data provide direct evidence that the expression of ASCs is enhanced in the acute stage of KD. After the application of IVIG, despite the level of CD138+ ASCs was not significantly higher than that in the acute stage (*P* = 0.6062, Fig. 1c), they maintained a relatively higher level (*P* = 0.0001, Fig. 1c), implying their responsibility for the humoral immunity in remission stage.

### The levels of memory B cells in different stages of KD

**Fig. 2:**
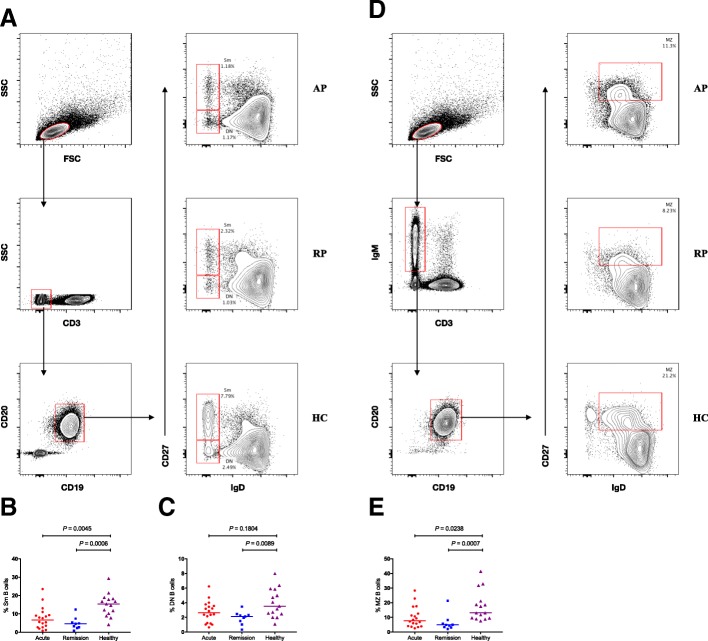
Flow cytometry analysis of the percentage of distinct subsets of memory B cells in KD patients. **a** Flow cytometry analysis of Sm and DN B cells. **b–c** Quantitative analysis of Sm and DN B cells. **d** Flow cytometry analysis of MZ B cells. **e** Quantitative analysis of MZ B cells. The data are represented as dot plots or are expressed as the mean percentage of antibody secreting cells of individuals. The horizontal lines stand for the median values. Sm represents switched memory B cells with phenotype CD27 + IgD-. DN represents double negative B cells with phenotype CD27-IgD-. MZ represents marginal zone B cells with phenotype CD27 + IgD + IgM+

As memory B cells are another important portion of the humoral immunity, we subsequently explored their overall levels in acute and remission stages of KD. PBMCs with immune-staining CD3, CD19, CD20, CD27, IgD and IgM were investigated. Upon these surface markers, memory B cells were initially categorized into two subgroups namely CD27 + IgD- (Switched memory, Sm) and CD27-IgD- (Double negative, DN) B cells (Fig. 2a) [22, 23]. Both of the two subsets were gated on the CD3-CD19 + CD20+ B cells. Whether in the acute stage or remission stage, the percentages of Sm B cells were significantly lower than that in HCs (*P* = 0.0045 and *P* = 0.0006, respectively; Fig. 2b). By contrast, the variation of DN B cells in the acute stage was not significant, whereas their level in remission stage was significantly lower than that in HCs (*P* = 0.1804 and *P* = 0.0089, respectively; Fig. 2c). Next, we examined another subset namely CD27 + IgD + IgM+ (marginal zone, MZ) B cells [27, 28]. MZ B cells were gated initially on living lymphocytes, and then on CD3-IgM+ cells, and finally on CD19 + CD20+ cells (Fig. 2d). The percentages of MZ B cells in both the acute stage and remission stage were significantly lower than that in HCs (*P* = 0.0238 and *P* = 0.0007, respectively; Fig. 2e). Our results showed a relatively low level of memory cells whether in the acute stage or the remission stage.

### The correlations among ASCs, memory B cells and laboratory findings

**Fig. 3:**
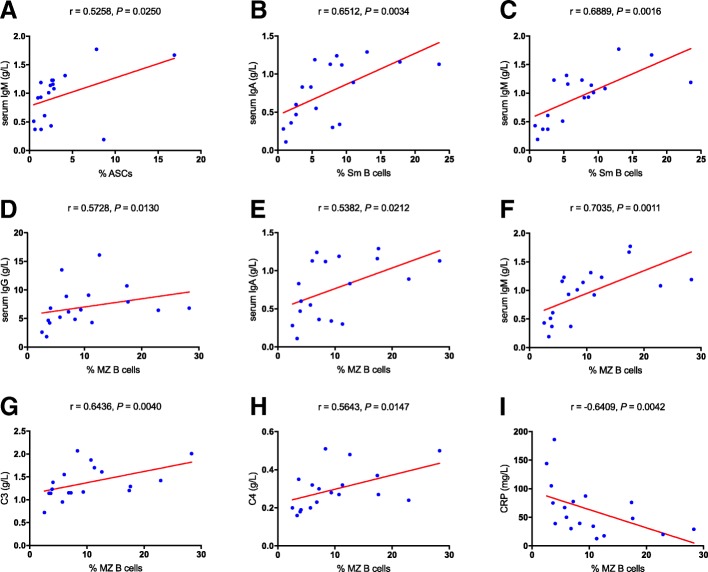
Correlation analysis in the acute stage of KD. **a–i** The correlations between either ASCs or memory B cells and laboratory tests including immunoglobulins (IgG, IgA, and IgM), CRP, C3, and C4. CRP, C-reactive proteins; C3, complement component 3; C4, complement component 4

**Fig. 4:**
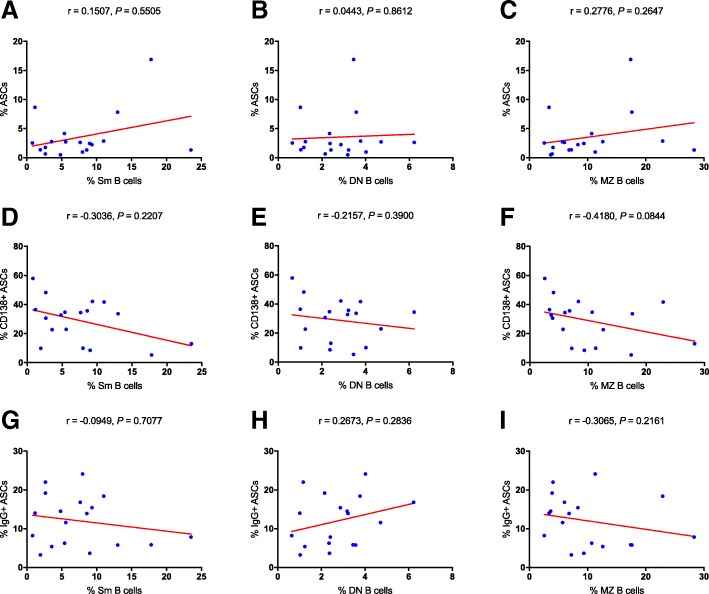
Correlation analysis in the acute stage of KD. **a–i** The correlations among ASCs, the subsets of ASCs and the subsets of memory B cells

With the purpose for further understanding the roles of ASCs and memory B cells in KD, we analyzed their correlations with laboratory findings including the values of CRP and ESR, the levels of serum immunoglobulins (IgG, IgA, IgM) and the levels of complement C3 and C4. The percentage of ASCs shown a positive correlation with the level of serum IgM (*r* = 0.5258, *P* = 0.0250, Fig. 3a). The percentage of Sm B cells was positively correlated with the levels of serum IgA and IgM (*r* = 0.6512, *P* = 0.0034, Fig. 3b; *r* = 0.6889, *P* = 0.0016, Fig. 3c; respectively). It is impressive that MZ B cells shown diverse correlations with both adaptive and innate immunity. First, the percentage of MZ B cells was positively correlated with the level of serum IgG, IgA and IgM (*r* = 0.5728, *P* = 0.0130, Fig. 3d; *r* = 0.5382, *P* = 0.0212, Fig. 3e; *r* = 0.7035, *P* = 0.0011, Fig. 3f; respectively). Second, we also found their positive correlations with the levels of complement C3 and C4 (*r* = 0.6436, *P* = 0.0040, Fig. 3g; *r* = 0.5643, *P* = 0.0147, Fig. 3h; respectively), and their negative correlation with the value of CRP (*r* = − 0.6409, *P* = 0.0042, Fig. 3i). Although the percentages of IgG+ ASCs and CD138+ ASCs were also increased in the acute stage, neither of them shown any significant correlations with laboratory findings (shown in Additional file 1: Figure S1). Due to the opposite variation trend between ASCs and memory B cells, we subsequently explored the correlations among subsets of ASCs and subsets of memory B cells. We did not find any significant correlations among them (Fig. 4a-i). Thus, ASCs and memory B cells maybe affect the development of KD in distinct ways. Besides, there is no absolute connection between the reduction of memory B cells and the elevation of ASCs.

### Variations of ASCs and memory B cells after IVIG administration

**Fig. 5:**
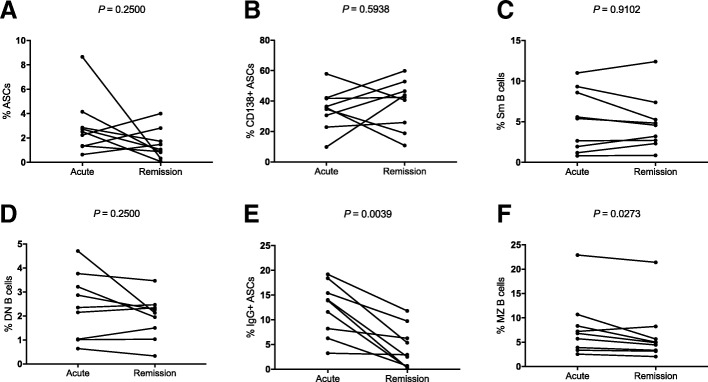
**a–f** The variations of ASCs and memory B cells in the remission stage

To present a more integrated status of humoral immunity during the course of KD, we investigated the variations of ASCs and memory B cells in the same individual at different stages. Among patients, we found heterogeneous variations in ASCs and CD138+ ASCs cells (*P* = 0.2500, Fig. 5a; *P* = 0.5938, Fig. 5b; respectively), as well as in Sm and DN B cells (*P* = 0.9102, Fig. 5c; *P* = 0.2500, Fig. 5d; respectively). By contrast, the percentage of IgG+ ASCs and the percentage of MZ B cells were significantly reduced in remission stage (*P* = 0.0039, Fig. 5e; *P* = 0.0273, Fig. 5f; respectively). It is of note that the percentage of MZ B cells, which has reduced in the acute stage, further decreased in the remission stage. Collectively, no matter it is on the overall or individual perspective, it can be concluded that the dysregulated humoral immunity might not be entirely restored in the remission stage.

## Discussion

Based on our observations, it can be concluded that ASCs were involved in the acute stage of KD. In this stage, we found a significantly increased percentage of ASCs. ASCs could be anti-inflammatory via the secretion of immunoglobulins, alternatively through the production of IL-10 [29]. So next, we investigated the correlations between the percentage of ASCs and laboratory findings including the levels of serum immunoglobulins and the inflammatory indicators. The percentage of ASCs positively correlated with the level of serum IgM, but not IgG or IgA, indicating that the ASCs played their roles in the acute stage of KD predominantly through secreting IgM. However, individuals with a higher percentage of ASCs did not show relatively lower levels of inflammatory indicators, suggesting that their anti-inflammatory role in the acute stage of KD was likely to be less satisfactory. Hence, it might be reasonable to speculate that these increased ASCs in the acute stage of KD developed from extrafollicular B cells [19, 30]. The increased percentage of ASCs may be as a result of the elevation of stimulative factors. One of the advantages for the differentiation of B cells into ASCs may be the elevation of B-cell-activating factor (BAFF), which can effectively promote the proliferation, differentiation, and survival of B cells [31]. Another superiority for ASCs is the increased levels of cytokines, such as IL-6, IL-17 and IL-21 [13, 32, 33]. Furthermore, increased expression of CD138 is capable of promoting the maturation, accumulation and particularly, survival of ASCs upon IL-6 signaling [34]. Therefore, elevated BAFF and CD138 in concert with those increased cytokines can directly enhance the differentiation of B cells into ASCs. Meanwhile, the expression of IgG in the cytoplasm was enhanced. In contrast to our results, *Shingadia* et al. have reported a decreased absolute number of cytoplastic IgG+ plasma cells [11]. The precise reason causing the contrary results is unclear. Perhaps, it is due to the difference of the definition of the ASCs. Recent research found that CD19 negative ASCs would emerge in circulation at the early stage plasmablasts to plasma cell transition [35]. Hence, the category of ASCs based on CD19 expression might leave out a part of B cell capable of producing antibodies. The enhanced expression of cytoplasmic IgG positive plasmablasts was demonstrated in ulcerative colitis likewise [36]. Their results also found positive correlations between the level of IgG+ plasmablasts and indicators of disease activity, thereby suggesting this subset could be pro-inflammatory in the pathogenesis of ulcerative colitis. In KD, the overall levels of both cytoplasmic IgG and inflammatory indicators were increased, apparently insisting the pro-inflammatory role of IgG+ ASCs. However, our data did not find any significant positive correlations between IgG+ ASCs and inflammatory indicators including CRP, ESR. Additionally, although the percentage of IgG+ ASCs in the acute stage was significantly increased, it was not correlated with the level of serum IgG, and the median level of serum IgG in KD patients was equivalent to that in HCs, suggesting the increased cytoplasmic IgG was not completely excreted. Indeed, it was reported that cytoplasmic IgG was likely to be beneficial for the elimination of kinds of intracellular virus, which were indicated as pathogenic candidate agents in KD, possibly via intracellular antibody-mediated degradation [37–39]. Thus, we hypothesize that the roles of cytoplasmic IgG in KD are heterogeneous and may be related to the kind of invading pathogen.

The precise mechanism of IVIG in the treatment of KD remains unknown. Potential mechanisms of action include the neutralization of toxin, modulation of the activity of monocyte/macrophage and neutrophils, provision of anti-idiotypic IgG, regulation of T cell differentiation and cytokine release [40]. To date, the study regarding the action of IVIG on B cells in KD was limited. A previous study found a significant decrease in B cells after IVIG treatment, suggesting that IVIG could restore B-cell abnormalities [41]. In the present study, we found the percentage of IgG+ ASCs, which was increased in the acute stage, was significantly reduced after IVIG administration. The results provided strong evidence demonstrating the involvement of IgG+ ASCs in KD inflammation and implied a regulatory effect of IVIG on IgG+ ASCs. The decrease of IgG in ASCs cytoplasm may be as a result of the increased level of serum IgG caused by the application of high dose IVIG, which contributes to the neutralization of toxin and antigens, and thereby negatively regulates the synthesis of cytoplasmic IgG. Those cell-penetrating ingredient antibodies in IVIG may be responsible for the inhibition of cell activation and the clearance of intracellular pathogens [42]. It also could be associated with the presence of anti-BAFF antibodies in IVIG preparation and the triggering of Fas apoptotic pathway by IVIG [43]. Moreover, it was shown that IVIG promoted the expression of Fc-gamma Receptor (FcγR)-IIB on B cells, which could bind to the Fc segment of IgG and subsequently induced inhibitory signal [44]. Thus, these mechanisms would eventually attenuate the activities of IgG+ ASCs and enhance their sensitivity to apoptosis. Importantly, our data did not find a definitely inhibitory effect of IVIG on ASCs or CD138+ ASCs, because they presented a heterogeneous variation after IVIG administration. Accordingly, the action of IVIG on ASCs should include other regulatory mechanisms. It was reported that the expression of A Proliferation-inducing Ligand (APRIL), which is advantageous for development and survival of B cells, was increased after IVIG administration, opposing to the variation of BAFF [31]. In addition, in vitro study on the patients with SLE found increased plasma cell differentiation in the presence of IVIG [45]. Consequently, the specific role of IVIG in regulating ASCs remains to be further elucidated. Another valuable matter was that in comparison with other ASCs, the overall level of CD138+ ASCs in remission was higher, despite not significantly, than that in acute, suggesting their distinct role in the remission stage of KD. The latest researches demonstrated that CD138+ plasma cells in bone marrow were inclusive of a group of long-lived plasma cells, which present a memory nature through persistent secretion of specific antibodies even though the patients had not exposed to the pathogens for decades [46–48]. Hence, it can be speculated that those increased CD138+ ASCs may be an explanation for the low recurrent rate of KD, as well as for the self-antibodies lasting for years [49].

Besides ASCs, memory B cells were also believed to be essential for maintaining humoral immunity. In the acute stage, the percentage of DN B cells was lower, but not significantly, than that in HCs. By contrast, the percentages of Sm and MZ B cells were significantly decreased. The data demonstrated that the patients with KD underwent profound variations and imbalances of memory B-cell subsets. Intriguingly, the variation of memory B cells was contrary to the variation of ASCs; however, there were no definite correlations among the subsets of memory B cells and ASCs. Thus, it may be hard to decide whether the reduction in memory B cells is simply due to their switching into ASCs. Among others, MZ B cells shown correlations with multiple laboratory findings, suggesting that MZ B cells contributed to both innate and adaptive responses, more likely, to the alleviation of inflammation via positive effects on immunoglobulins secretion and complements activation. A systemic review of the distinct features of MZ B cells insisted on their importance in inflammation [50]. When patients entered into remission, the overall levels of memory B cells were significantly lower than those in HC. It seemed that suppressed memory response was throughout the course of KD and IVIG failed to modulate the memory immunity. However, it may be partial to draw this conclusion only upon the analysis of circulating memory B cells because in some conditions, memory B cells are abundant in the spleen [51, 52] or the mucosa [53]. Therefore, in order to present a more integrated memory immunity, it may be necessary to investigate the status of memory B cells in the organ or tissue.

In our current study, we described a general picture of the status of ASCs and memory B cells during the course of KD. However, we also realized the limitations of our study. First, it may be worthy of analyzing the function of ASCs, particularly IgG+ ASCs, as well as those long-lived ASCs in bone marrow, if accessible. Second, investigation on the memory response in such as spleen and mucosa lymphoid tissue may be necessary. Third, the sample size in the remission stage should be enlarged. We will focus on these issues in subsequent studies.

## Conclusions

Our data directly demonstrated the involvement of ASCs in KD. Among the subsets, the significant expansion of IgG+ ASCs in the acute stage indicated their importance in KD inflammation. Besides, IgG+ ASCs reduced consistently after IVIG administration, suggesting that IVIG played a role in inhibiting the expression of cytoplasmic IgG, which might be one of the mechanisms of action of IVIG in treatment of KD. We also firstly described memory B cells in this disease, the level of which was relatively low in both acute and remission stages. Our study would be beneficial for the further understanding of the pathogenesis of KD.

## Additional file

Additional file 1: **Figure S1.** Correlation analysis. (A) Correlations between the percentage of IgG+ ASCs and laboratory findings including CRP, ESR, immunoglobulins, and complement C3 and C4. (B) Correlations between the percentage of CD138+ ASCs and those laboratory findings. (PDF 69 kb)

## Data Availability

The datasets used and analyzed during the current study are available from the corresponding author on reasonable request.

## Abbreviations

ASCs: Antibody secreting cells
DN: Double negative
GC: Germinal center
HCs: Healthy controls
IVIG: Intravenous immunoglobulin
KD: Kawasaki disease
MZ: Marginal zone
PBMC: Peripheral blood mononuclear cell
Sm: Switched memory
