# Supplementary figures and images for: Distinct variations of antibody secreting cells and memory B cells during the course of Kawasaki disease
Source: BMC Immunol. 2019 Jun 3;20:16. doi: 10.1186/s12865-019-0299-7 (PMC6547606; doi:10.1186/s12865-019-0299-7)

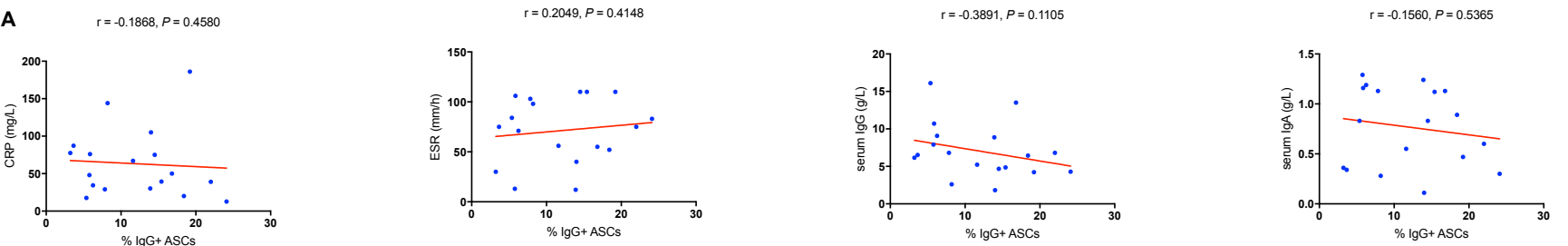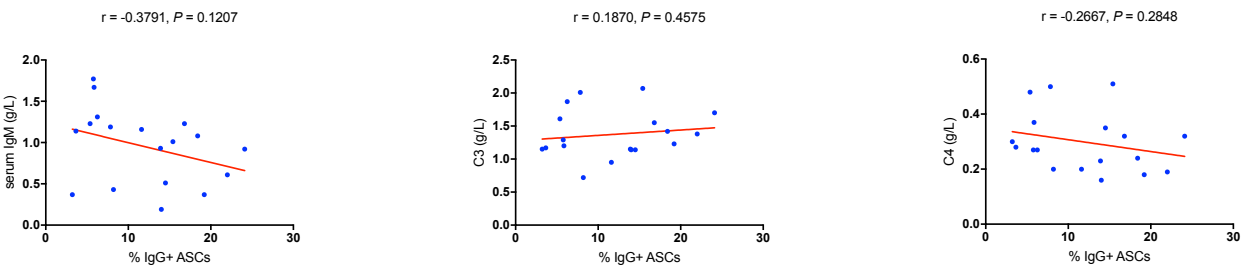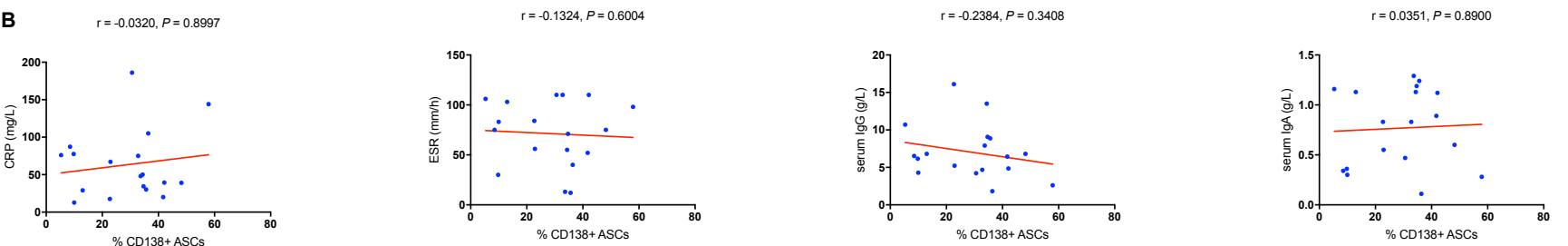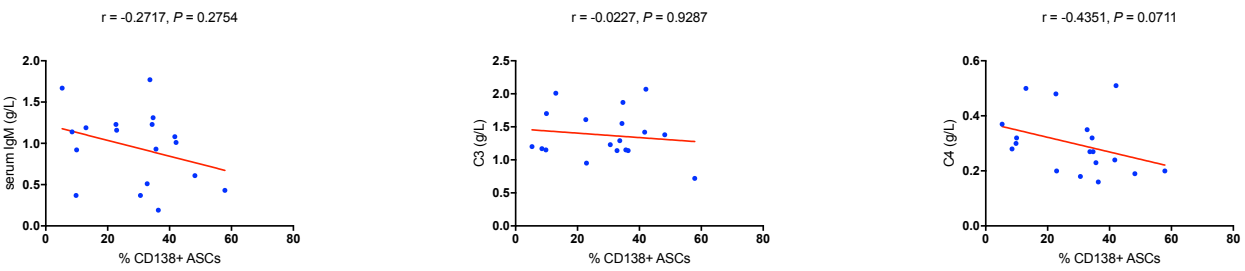

Supplement: Supplementary file 1 — Figure S1. Correlation analysis. (A) Correlations between the percentage of IgG+ ASCs and laboratory findings including CRP, ESR, immunoglobulins, and complement C3 and C4. (B) Correlations between the percentage of CD138+ ASCs and those laboratory findings. (PDF 69 kb) [file 12865_2019_299_MOESM1_ESM.pdf]
